# COVID-19 Vaccination Coverage in Patients with Rheumatic Diseases in a German Outpatient Clinic: An Observational Study

**DOI:** 10.3390/vaccines10020253

**Published:** 2022-02-07

**Authors:** Marco Krasselt, Christoph Baerwald, Olga Seifert

**Affiliations:** Rheumatology Unit, Clinic for Endocrinology, Nephrology and Rheumatology, Department of Internal Medicine, Neurology and Dermatology, University of Leipzig, Liebigstr. 20/22, 04103 Leipzig, Germany; christoph.baerwald@medizin.uni-leipzig.de (C.B.); olga.malysheva@medizin.uni-leipzig.de (O.S.)

**Keywords:** SARS-CoV-2, COVID-19, vaccination, vaccination rate

## Abstract

Background: In the second year of the COVID-19 pandemic, highly effective and safe vaccines became available. Since patients with rheumatic diseases show increased susceptibility to infections and typical medications raise the risk of severe COVID-19, high vaccination coverage is of significant importance to these patients. Methods: Consecutive patients with different rheumatic diseases were asked for their vaccination status regarding COVID-19, influenza and Streptococcus pneumoniae during their routine consultations. Any reported vaccination was validated with their personal vaccination card and/or by reviewing the CovPass smartphone app. Reasons for not having a COVID-19 vaccination were documented. Results: A total of 201 patients (mean age 62.3 ± 14.1 years) were included, the majority of them (44.3%) with rheumatoid arthritis, followed by spondyloarthritis (27.4%) and connective tissue diseases (21.4%). Vaccination coverage for SARS-CoV-2 was 80.1%; 85.6% got at least the first vaccination shot. Both valid influenza and pneumococcus coverage were associated with a higher probability of SARS-CoV-2 vaccination (odds ratio (OR) 6.243, 95% confidence interval (CI) 2.637–14.783, *p* < 0.0001 and OR 6.372, 95% CI 2.105–19.282, *p* = 0.0003, respectively). The main reason for a missing SARS-CoV-2 vaccination (70%) was being sceptical about the vaccine itself (i.e., the subjective impression that the vaccine was not properly tested and fear of unwanted side effects). Conclusions: Vaccination coverage against SARS-CoV-2 is high in patients with rheumatic diseases. Nevertheless, there are unmet needs regarding vaccination education to further increase vaccination rates.

## 1. Introduction

The COVID-19 pandemic puts people with rheumatic diseases at an increased risk since they are highly susceptible to infections. During the last year, typical rheumatologic therapies such as high-dose glucocorticoids and the CD20-depleting agent rituximab have been shown to predispose patients to severe COVID-19 [1,2,3]. Consequently, proper vaccination against COVID-19, caused by the severe acute respiratory syndrome coronavirus type 2 (SARS-CoV-2), is of particular importance among patients with rheumatic diseases. The German nationwide vaccination programme started at the end of 2020, on 27 December [4], and to date, four vaccines (two mRNA-based, two vector-based) have been approved for application [5]. They have been demonstrated to be both safe and effective in the general population [6,7,8,9].

COVID-19 vaccination is recommended for patients with rheumatic diseases by the Standing Committee on Vaccination (STIKO) of the Robert Koch Institute (RKI) as well as the German Society for Rheumatology (DGRh) [10,11].

Contrary to the well-known COVID-19 vaccination rates in the general population, details on vaccination coverage in people with rheumatic diseases are scarce. The primary aim of this study was, therefore, to measure vaccination coverage of COVID-19 in a German cohort of patients with rheumatic diseases. To gain more insight into individual vaccination behaviour and confidence, current influenza and pneumococcal protection were evaluated. Finally, reasons for vaccination hesitancy have been documented.

## 2. Materials and Methods

Consecutive adult patients with a rheumatic disease were recruited from our outpatient clinic during their regular consultations between June and August 2021. They were asked for their vaccination status regarding SARS-CoV-2, influenza (last year, 2020) and Streptococcus pneumoniae (within the last five years). Vaccines were typically administered by general practitioners or in vaccination centres. None were applied in our outpatient clinic. Any reported vaccination was double-checked by either reviewing the individual vaccination cards or using the CovPass smartphone app. CovPass is an application from the Robert Koch Institute for German residents, which allows a European Union Digital COVID Certificate for their valid SARS-CoV-2 vaccination to be stored and recalled [12]. Approved vaccines of any manufacturer (i.e., Comirnaty (Biontech/Pfizer), Spikevax (Moderna), Vaxzevria (AstraZeneca) and COVID-19 vaccine Janssen (Janssen-Cilag/Johnson & Johnson)) were considered. The reasons for not having a vaccination were documented. Patients’ demographic characteristics were obtained from the medical record.

The design of the study was approved by the ethics committee of the University of Leipzig (282/21-ek), and informed consent was obtained from all participants before study enrolment.

To describe continuous data, mean and standard deviation (SD) were used. Categorical data were described with absolute or relative frequencies. To compare the frequencies of categorical variables, Chi-squared tests were performed. A significant statistical difference was assumed when the *p*-value was <0.05. All analyses were two-tailed and conducted using GraphPad PRISM Version 8 for Mac (GraphPad Software Inc., San Diego, CA, USA).

## 3. Results

In total, *n* = 201 patients with different rheumatic diseases (mean age 62.2 ± 14.1 years, see Table 1) were included in this study. Out of 191 patients (85.6%), 172 had at least one vaccination against SARS-CoV-2. Overall, 161 patients were fully vaccinated (i.e., two vaccination shots) (80.1%). In patients ≥60 years, the vaccination rate (at least one shot) was higher compared to patients <60 years (90.1 vs. 78.8, *p* = 0.025).

### 3.1. Vaccination Coverage Is Higher in Patients with Either Current Influenza or Pneumococcus Vaccination

**Figure 1:**
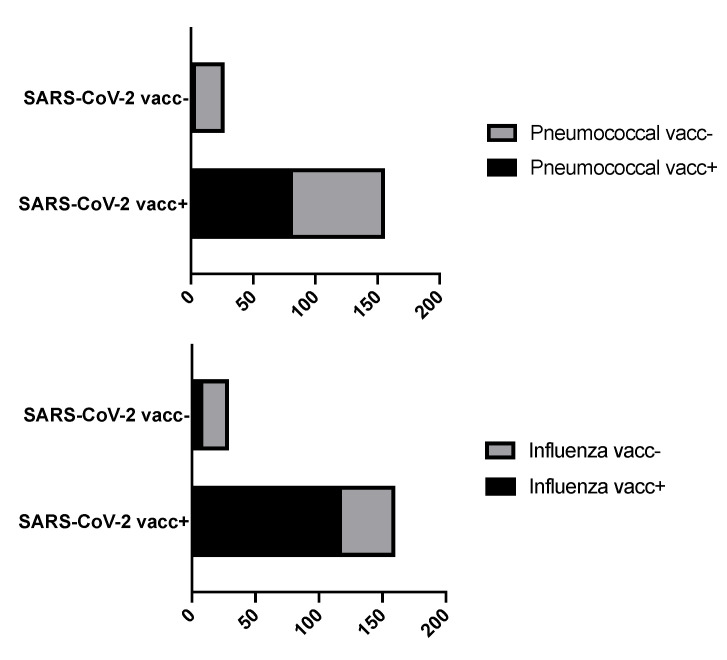
Vaccination coverage for SARS-CoV-2 is increased in patients with both current influenza and pneumococcal vaccination.

The probability of being vaccinated against SARS-CoV-2 was markedly higher among patients with a valid influenza vaccination in 2020 (odds ratio (OR) 6.243, 95% confidence interval (CI) 2.637–14.783, *p* < 0.0001). For patients with a valid pneumococcal vaccination, we saw a similar relationship (OR 6.372, 95% CI 2.105–19.282, *p* = 0.0003); see Figure 1. Neither the underlying rheumatic disease nor the used medication had a significant impact on vaccination coverage (data not shown).

### 3.2. Safety Concerns Are the Main Reason for Non-Vaccination

**Figure 2:**
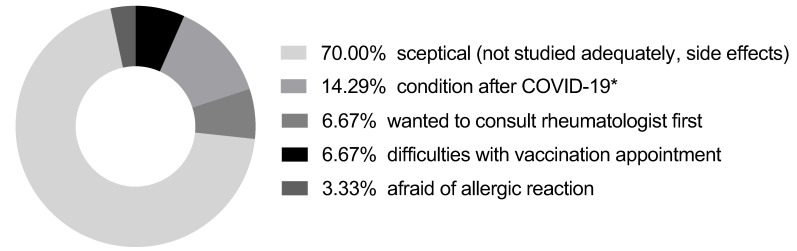
Reasons for non-vaccination, *n* = 29. *—For people after symptomatic COVID-19 infection, a single-shot vaccination 6 months afterward is recommended [10].

When asking patients for their motives for not being vaccinated, scepticism regarding the used vaccines and fear of unwanted side effects was found to be the main reason (70%, see Figure 1). This particularly included potential side effects and the subjective impression that the vaccines might not have been tested properly yet. Interestingly, no patient hesitated with vaccination because of caveats regarding their individual antirheumatic medication. Some patients (14.29%) have had symptomatic COVID-19 infection, and vaccination is not recommended earlier than 6 months afterward. A few patients (6.9%) missed their outpatient appointment at the beginning of the year and wanted to talk to the rheumatologist in person before getting the vaccine. Other reasons (e.g., difficulties with vaccination appointments, see Figure 2) were less relevant. One patient had a known history of a severe allergic reaction to an influenza vaccine and therefore rejected a SARS-CoV-2 vaccination.

## 4. Discussion

The main outcome of our study is the high vaccination rate against SARS-CoV-2 in a German cohort with inflammatory rheumatic diseases. Of importance, vaccination was more likely in patients with either a valid influenza or pneumococcus vaccination and in patients 60 years of age or older.

An Australian online survey among patients with rheumatic diseases regarding vaccination willingness reported that 65% were willing to have a COVID-19 vaccine [13]. While the actual vaccination rate is markedly higher in our cohort (80.1%), the online survey also showed that willingness was higher in patients with recent influenza and pneumococcal vaccination (OR 2.69) [13]. This finding was confirmed in our investigation, showing even higher odds (OR 6.372 for pneumococcal vaccination). Both vaccines are recommended for patients with rheumatic diseases [14]. A recent survey among >200 German rheumatologists reported that 99% would recommend a COVID-19 vaccination to their patients [15]. This recommendation follows the guideline of the German Society for Rheumatology (DGRh), which also includes booster vaccinations by now [11].

Our study collected data in the middle of 2021. In the beginning of 2021, COVID-19 vaccination was recommended verbally to all our patients during their outpatient consultations. This might have contributed to the high vaccination rate of 80% we observed. The current vaccination rates for the general population in Germany as of September 9th were 66.3% (at least one shot) and 61.9% (complete vaccination) [16]. A French study found an overall SARS-CoV-2 vaccination rate of 14.5% in a cohort of patients with autoimmune and inflammatory diseases (mostly patients with SLE and vasculitis) at the end of March 2021. This rate could be boosted to 64.6% by an investigator-initiated vaccination programme [17]. An Australian investigation in tumor patients reported a comparably low vaccination rate of 65.2% as of August 2021 [18]. Colleagues from Germany actively offered a vaccination to hearth transplant patients and reached a vaccination rate of 71% [19]. In German healthcare workers, the vaccination rate was 62% in spring 2021, with a further 22% who wanted to be vaccinated as soon as possible [20]. Compared to other vaccines (e.g., tetanus, pertussis, but also pneumococcal and influenza vaccination [21,22,23]), the vaccination rate against SARS-CoV-2 we found in our cohort (80.1%) is pretty high. This is most likely attributable to the public and medical attention the COVID-19 pandemic generates.

The association between an increased age (≥60 years) and probability of SARS-CoV-2 vaccination most likely is a result of the vaccine shortage at the beginning of 2021: German recommendations consequently stratified vaccination priority according to risk, and therefore, older people got vaccinated first (vaccination prioritizing was cancelled in June 2021 [24]). On the other hand, vaccination awareness in general is higher in older patients.

The main patient concern regarding the COVID-19 vaccination was that the vaccines might not have been studied properly, so some patients feared unwanted side effects. This finding is in line with an investigation in cancer patients [18]. Accordingly, in a cohort of patients with autoimmune and inflammatory diseases, the main reason to refuse vaccination was the fear of long-term adverse events [17]. At first glance, this is somewhat puzzling, since the available vaccines have been studied extensively [6,7,8,9], demonstrating not only high efficacy but also safety. Some of the patient concerns might have arisen from the rare occurrence (approx. 8.1 per million after the first shot, 2.3 per million after the second shot) of thrombosis with thrombocytopenia syndrome (TTS) after vaccination with the vector-based vaccine Vaxzevria (AstraZeneca) [25,26]. For the mRNA vaccines, self-limiting myocarditis and pericarditis have been reported (approx. 10 and 18 per million, respectively) [27]. Another reason might be the ongoing debate about the new technology, particularly with regard to the mRNA-based vaccines (alleged “gene therapy”). The significant association between preceding influenza or pneumococcal vaccination and protection against COVID-19 might imply vaccine scepticism in general. Ongoing patient education is clearly needed to improve vaccination rates in patients at risk.

The main limitation of our investigation is the tertiary character of our university hospital with specialized rheumatologic care. The findings in our study might therefore not be generalizable to standard rheumatologic care settings. Nonetheless, we believe that our finding of a high vaccination rate among patients with rheumatic diseases is encouraging and contributes greatly to our current knowledge regarding COVID vaccination.

## 5. Conclusions

Vaccination rates against SARS-CoV-2 are fortunately high in our cohort of patients with rheumatic diseases in a tertiary university hospital. Scepticism regarding the safety of the approved vaccines was the main reason for not being vaccinated in our cohort. This finding emphasizes the importance of patient education with particular attention to the safety of the available COVID-19 vaccines. Preferably, education should be ensured by both rheumatologists and general practitioners to further improve vaccination rates.

## Figures and Tables

**Table 1:** Clinical characteristics of the included patients (*n* = 201). Numbers are shown as % or mean with standard deviation (SD).

Characteristics	Result (*n* = 201)	SARS-CoV-2 Vaccination Rate
Mean age, years	62.2 ± 14.1	n.a.
Female, *n* (%)	133 (66.2)	110 (82.7)
Patients ≥ 60 years, *n* (%)	121 (60.2)	109 (90.1)
SARS-CoV-2 vaccination (complete), *n* (%)	161 (80.1)	n.a.
SARS-CoV-2 vaccination (at least one shot), *n* (%)	172 (85.6)	n.a.
Influenza vaccination 2020, *n* (%)	127 (63.2)	118 (92.9)
Pneumococcal vaccination in the last 5 years, *n* (%)	86 (42.8)	82 (95.3)
Rheumatic disease, *n* (%)		
Rheumatoid arthritis	89 (44.3)	74 (83.1)
Spondyloarthritis ^1^	55 (27.4)	51 (92.7)
Connective tissue diseases	41 (20.4)	33 (80.5)
Systemic lupus erythematosus	34 (82.9)	27 (79.4)
ANCA-associated vasculitis	11 (5.5)	10 (90.9)
Idiopathic juvenile arthritis	3 (1.5)	2 (66.7)
Large-vessel vasculitis	1 (0.5)	1 (100)
Adult-onset Still’s disease	1 (0.5)	1 (100)

^1^—Including axial spondyloarthritis and psoriatic arthritis.

## Data Availability

The data presented in this study are available on reasonable request from the corresponding author. The data are not publicly available due to privacy reasons.
